# An improved behavioural assay demonstrates that ultrasound vocalizations constitute a reliable indicator of chronic cancer pain and neuropathic pain

**DOI:** 10.1186/1744-8069-6-18

**Published:** 2010-03-26

**Authors:** Martina Kurejova, Ulrike Nattenmüller, Ullrich Hildebrandt, Deepitha Selvaraj, Sebastian Stösser, Rohini Kuner

**Affiliations:** 1 Institute for Pharmacology, University of Heidelberg, Im Neuenheimer Feld, Heidelberg, 69120 Germany

## Abstract

**Background:**

On-going pain is one of the most debilitating symptoms associated with a variety of chronic pain disorders. An understanding of mechanisms underlying on-going pain, i.e. stimulus-independent pain has been hampered so far by a lack of behavioural parameters which enable studying it in experimental animals. Ultrasound vocalizations (USVs) have been proposed to correlate with pain evoked by an acute activation of nociceptors. However, literature on the utility of USVs as an indicator of chronic pain is very controversial. A majority of these inconsistencies arise from parameters confounding behavioural experiments, which include novelty, fear and stress due to restrain, amongst others.

**Results:**

We have developed an improved assay which overcomes these confounding factors and enables studying USVs in freely moving mice repetitively over several weeks. Using this improved assay, we report here that USVs increase significantly in mice with bone metastases-induced cancer pain or neuropathic pain for several weeks, in comparison to sham-treated mice. Importantly, analgesic drugs which are known to alleviate tumour pain or neuropathic pain in human patients significantly reduce USVs as well as mechanical allodynia in corresponding mouse models.

**Conclusions:**

We show that studying USVs and mechanical allodynia in the same cohort of mice enables comparing the temporal progression of on-going pain (i.e. stimulus-independent pain) and stimulus-evoked pain in these clinically highly-relevant forms of chronic pain.

## Finding

On-going, long-lasting pain is a classical feature of cancer and neuropathic pain. Various types of carcinomas and sarcomas that metastasize to skeletal bones cause hyperalgesia, i.e. exaggerated pain in response to nociceptive stimuli as well as on-going, unstimulated pain [1,2]. Clinical features of neuropathic pain also include on-going pain [3,4]. In a majority of studies, evoked motor activities, such as licking, shaking, lifting or withdrawal of the affected body region, have been used as indications of disease-induced evoked hypersensitivity. In contrast, the analysis of on-going pain has remained a challenge. A very recent study has described the utility of analgesic drug-induced conditioned place preference as an indicator of on-going pain in neuropathic rats, which can be used to test the effects of novel, putative analgesic drugs on spontaneous pain in rats [5].

Ultrasound vocalizations (USVs) have been previously postulated to be an indicator of on-going pain and have been proposed as a potential method for measuring the negative affective components of pain. However, convincing experimental evidence has only come from experiments recording USVs evoked acutely upon activation of nociceptive neurons, e.g. application of nociceptive irritants, such as capsaicin and formalin, or acute nociceptive stimuli, such as pinch or incision of the skin [6-9]. Martino et al. [10] showed that the central inflammation caused by intracerebroventricular injection of lipopolysaccharide increases USVs in rats. However, other studies have arrived at different conclusions and raised doubts about the relevance of USVs to pain [11-13]. Importantly, several previous studies have failed to detect increased USVs in models of persistent or chronic pain [11-13]. Numerous factors can confound the results obtained in behavioural analyses of USVs and obfuscate inferences as to whether persistent pain states are associated with change in USV patterns. For example, in several studies mice were immobilized in the vicinity of a detector in a restrained state (e.g. [8]), thereby raising the possibility of involvement of a large stress component. Other studies analyzed USVs at a frequency of 22 kHz (e.g. [12,14]), which are known to be associated with alarm cries in rodents as a part of a defence pattern in response to a predator [15]. This can seriously confound observations when several animals are tested in the presence of each other during analyses of USVs in pain models. Furthermore, USVs have been associated with a fear response [14], novelty-induced alarm and anxiety [16], as well as mating and social calls, showing that USVs can be generated as by-products of locomotor and exploratory activity of rodents as well as novelty or predator-induced fear during behavioural analyses. Furthermore, the level of background noise and the ultrasonic frequency used further represent decisive factors in these analyses. We therefore set out to systematically address these confounding factors in an effort to develop an assay which would enables delineating whether USVs patterns change in chronic pain states associated with on-going pain. As models, we employed spared nerve injury-induced neuropathic pain in mice (SNI) of the C57BL6, and bone metastases-induced cancer pain in mice of C3H/HeNCrl background.

As a first step, we developed a chamber with an improved design so as to allow analyses of USVs in unrestrained freely-moving mice. The mice were placed in a custom-designed recording chamber made of plexiglass of the dimensions 23 × 17 × 14 cm. The inner walls of the chamber were covered with a 1 cm thick layer of Sylgard to prevent recording of sounds made by animals during locomotor activity or during exploration of the walls of the chamber. The measuring chamber was covered with a plexiglass lid fitted with two mini-3 bat, built-in, ultrasound detectors, each of them set to detect a different frequency. The recording of USVs was done using an automated system (Ultravox, Noldus Technology), consisting of an audio filter and an ADA-D converter and a computer with an analysis software (Ultravox 2.0, Noldus Technology). Because it is known that sonic vocalizations are not elicited in the absence of evoked pain [8], we only focused on the analyses of USVs in an effort to quantify ongoing pain in the absence of an acute pain stimulus. A trained observer conducted analyses in order to closely monitor behavioural patterns of the test animals. Tests were done online; however, the sound-attenuated containment chamber was also fitted with a video camera to permit later offline analyses. Based upon previous studies which have demonstrated the occurrence of USVs following acute application of nociceptive stimuli [6,8] we chose 37 kHz and 50 kHz as recording frequencies; however, we avoided 22 kHz, since it is associated with alarm cries [14,17]. Simultaneous monitoring of behavioural patterns and USVs enabled filtering out time periods over which mice indulged in activities which disturb USV patterns, such as walking, scratching or rearing along the chamber walls.

In addition to the modifications described above, we identified the following as interfering parameters, which may have potentially confounded results from previous studies: analyzing two mice at the same time (confrontation, fear), number of detectors (interference was seen when 4 detectors were switched on at the same time), detector settings (when sensitivity levels were set high, noise at audible frequencies was heard, which likely leads to stress in the test animals).

**Figure 1:**
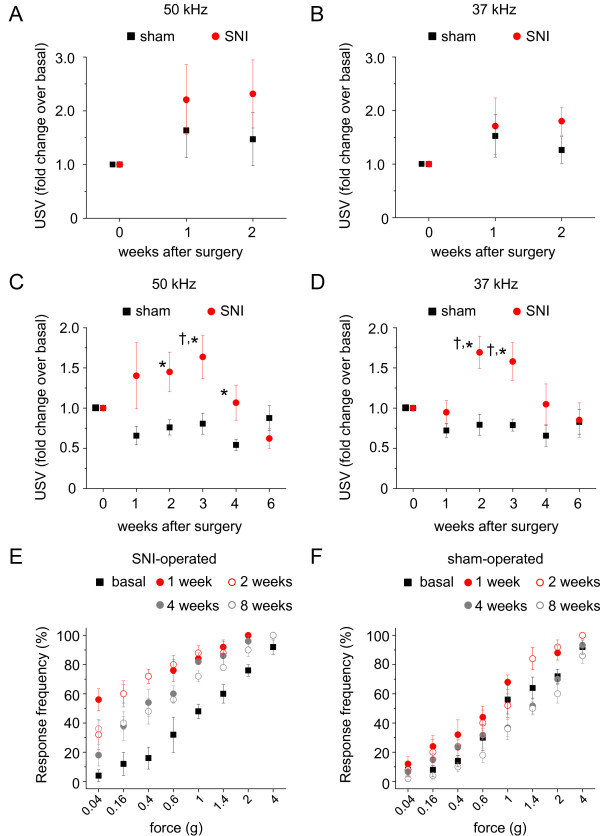
**Analysis of ultrasound vocalizations (USVs) and evoked mechanical hypersensitivity following spared nerve injury (SNI)**. C57Bl6 wild-type mice after SNI or sham treatment were tested at 50 kHz or 37 kHz recording frequencies. SNI- or sham-operated mice which had not been acclimatized to the setup did not show differences in USVs (A and B) (n = 9 mice/group). SNI- or sham-operated mice which had been pre-acclimatized to the recording chamber (C and D) showed differences in USV rate over basal state (pre-surgery values; †, p < 0.05) or with respect to each other (*, p < 0.05); ANOVA followed by post-hoc Fisher's test (n = 5 mice/group). Paw withdrawal responses to graded pressure applied via von Frey hairs prior to and following SNI (E) or sham treatment (F) are shown (n = 5 mice/group).

By eliminating the above confounding factors and by performing experiments in a secluded room with minimum background noise, using the custom-made chamber as described above, we were able to obtain very stable and reproducible patterns of USVs in mice. However, when mice are put in the recording chamber for the first time, which represents a novel and therefore potentially dangerous environment for the animal, one would still expect a fear component to the USV patterns obtained. We tested this notion by comparing USVs in unacclimatized mice with mice which had been acclimatized in the recording chamber for 3 times over duration of 15 min each 1 day prior to the actual measurements. Moreover, on the day of testing, the mice were also acclimatized in the chamber for 10 min prior to the actual measurement. Experiments showed that acclimatization to the recording chamber is a very important factor determining the outcome of USV patterns in neuropathic pain states. Mice which had not been acclimatized showed increased USVs at 1 and 2 weeks after sham surgery as compared to pre-surgery values at 50 kHz or 37 kHz recording frequencies (Fig. 1A and 1B). In contrast, sham-operated animals which had been acclimatized to the recording chamber showed a decrease in the rate of USVs in comparison to basal values at both recording frequencies (Fig. 1C and 1D; p < 0.05, ANOVA, Fisher's test). Interestingly, SNI-treated animals which had been acclimatized showed increased USVs over the 2^nd^, 3^rd ^and 4^th ^weeks following surgery in comparison to sham-treated animals and corresponding basal (p < 0.05 at 37 kHz and/or 50 kHz; Fig. 1C and 1D; p < 0.05, ANOVA, Fisher's test). At 6 weeks following SNI, the number of USVs was not statistically significantly different from the basal conditions or from sham-treated animals (Fig. 1C and 1D). These results show that following SNI-induced neuropathy, mice show enhanced USVs as compared to sham-treated animals in the absence of an acute pain stimulus, thereby indicating ongoing pain.

We also monitored the development of evoked mechanical hypersensitivity, in response to von Frey hair application to the plantar surface of the hind paw over the entire time period over which the animals had been analyzed with respect to USVs. This enabled directly comparing the development and temporal progression of evoked hypersensitivity and ongoing pain following neuropathy. Animals displayed marked hypersensitivity to low-threshold mechanical inputs at 1 and 2 weeks following SNI (p < 0.05; ANOVA followed by post-hoc Fisher's test). At 4-8 weeks following SNI, the degree of hypersensitivity was reduced but still significantly higher over basal values in SNI-treated animals (Fig. 1E). In contrast, sham-operated animals did not show any significant changes in mechanical sensitivity to von Frey input (Fig. 1F). Thus, comparing evoked mechanical sensitivity and USVs in the same cohort of animals enables us to infer that ongoing pain following SNI is of a shorter duration (up to 3 weeks after SNI) as compared to mechanical allodynia (more than 8 weeks after SNI).

**Figure 2:**
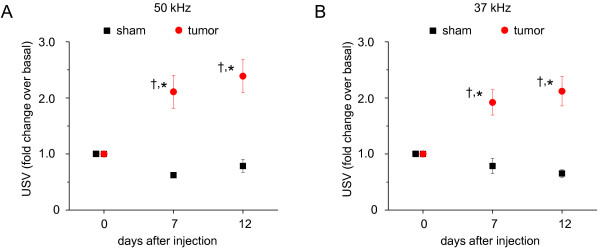
**Analysis of ultrasound vocalizations (USVs) following implantation of sarcoma cells**. C3H/HeNCrl wild-type mice injected with sarcoma cells (tumour) or PBS (sham) in the heel bone were tested at 50 kHz (A) or 37 kHz (B) recording frequencies. Tumour-bearing mice which had been pre-acclimatized to the recording chamber showed differences in USV rate over basal state (pre-surgery values; †, p < 0.05) or over sham-treated mice (*, p < 0.05); ANOVA followed by post-hoc Fisher's test (n = 9 per group).

We then asked whether USVs could function as an indicator of ongoing pain in cancer states, particularly following bone metastases. A model involving injection of sarcoma cells in the marrow of the calcaneus heel bone in mice has been previously shown to closely parallel bone cancer-evoked pain in humans [18]. In this model we observed that at 7 days following injection of tumour cells, mice displayed a doubling of the rate of USVs, which was further increased to 2.5 times at 12 days after injection at 50 and 37 kHz (Fig. 2A and 2B). In contrast, sham-treated mice (PBS injection in the calcaneus bone) either demonstrate a decrease over basal at 50 kHz or no change as compared to the basal values at 37 kHz (Fig. 2A and 2B, p < 0.05 as compared to tumour-injected animals, ANOVA followed by post-hoc Fisher's test).

**Figure 3:**
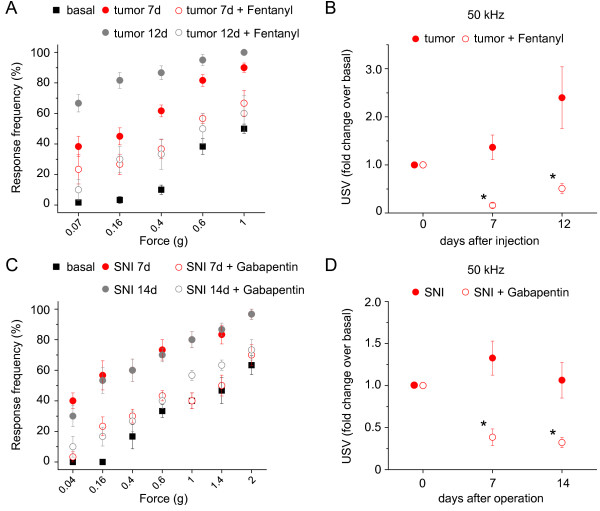
**Analysis of the effects of analgesics on ultrasound vocalizations (USVs) and evoked mechanical hypersensitivity**. (A and B) C3H/HeNCrl mice following implantation of sarcoma cells (tumour) in the heel bone were sub-cutaneously injected with fentanyl (0.1 mg/kg) or vehicle 15 min prior to the measurement of USVs (50 kHz) or von Frey responses (n = 6 mice/group). (C and D) SNI-treated C57Bl6 mice were injected intraperitoneally with gabapentin (30 mg/kg) or vehicle 1 h prior to measurement of USVs (50 kHz) or von Frey responses applied to the plantar surface (n = 6 mice/group). * denoted p < 0.05 as compared to drug-treated group at the corresponding time points after SNI/tumour implantation; ANOVA followed by post-hoc Fisher's test.

We then performed experiments addressing the time-course over which USVs increase and mechanical allodynia develops in the same cohort of mice. In our experience, depending upon experimental conditions pertaining to the injection sarcoma cells into the bone cavity and the growth curve of the sarcoma cells, the onset of significant tumour-induced mechanical allodynia can vary between 4-7 days [18,19]. In the experiments represented in Fig. 3A, significant mechanical allodynia was observed in the tumour group at day 7 after tumour implantation, which increased further in magnitude at day 12. Interestingly, tumour-injected mice showed a significant increase in USVs at day 7 after tumour implantation, which increased further in magnitude at day 12 (Fig. 3B). These results indicate that USV closely paralleled the time frame of mechanical allodynia in this model of bone cancer pain. Taken together with results from similar experiments performed in the SNI model, these results suggest that an increase in USVs in the SNI model of neuropathic pain is short-lived as compared to the persistent increase in USVs in the tumour-pain model. The duration of on-going pain may differ between these models, given the inherent differences in their etiology and pathophysiological mechanisms. It is noteworthy that mechanical allodynia in the tumour model is also significantly stronger in magnitude than in the SNI model and stays permanently until the mice are sacriffied [18,19], in contrast to SNI-induced mechanical allodynia which is reversible [20]. Strong on-going pain which progressively increases in magnitude with tumour growth is a hallmark of human bone metastases-induced pain [1,2]. Indeed, the observation that USVs progressively continue to increase over time after tumour implantation supports the notion that they represent a reasonable correlate for on-going pain.

In order to further study the association between disease-induced increase in USVs and on-going pain and its comparison to the course of mechanical allodynia, we studied the effects of clinically efficacious analgesic drugs on USV and mechanical allodynia behaviour. Fentanyl, an opioid which is clinically efficacious in alleviating tumour-induced on-going pain (e.g. [21]), has been shown to reduce mechanical allodynia in mouse models of cancer pain (e.g. [22]). We observed that an acute systemic administration of fentanyl significantly reduced tumour-induced mechanical hypersensitivity (Fig. 3A) and also completely reversed tumour-induced increase in USVs (50 kHz) at 7 and 12 days after tumour cell implantation (Fig. 3B; p < 0.05). Indeed, in the presence of fentanyl, tumour-bearing mice showed a USV pattern similar to that shown by sham-treated mice. Similarly, gabapentin, which is used in the clinical treatment of neuropathic pain syndromes (e.g. [23]) and has been shown to be efficacious in mouse models of neuropathic pain [24], inhibited SNI-induced mechanical allodynia and concurrently reversed SNI-induced increase in USVs (50 kHz) when tested at 1 and 2 weeks following SNI (Fig. 3C and 3D; p < 0.05). These results consolidate the notion that tumour/neuropathy-induced increase in USVs is a reliable correlate of on-going pain in these disease states.

These results show that factors such as restraint, stress, fear response, novelty-induced alarm calls, mating and social calls, amongst others confound the behavioural analysis of USVs in states of persistent, on-going pain. Upon overcoming these factors (at least partially), we observed that mice showing mechanical allodynia in neuropathic- and cancer pain states show a marked increase in USVs in the absence of externally applied stimuli. Furthermore, production of background noise, choice of frequency of recordings and reduction of interference arising from usage of multiple detectors during recordings were found to be important. Our results show that upon usage of an improved protocol as described above, USVs indicate a reliable, objective and measurable parameter for ongoing pain in neuropathic and cancer states. In the present study as USVs could be reliably studied in laboratory mice, which, albeit challenging to study in behavioural paradigms, are very important from the perspective of genetic and molecular studies. We believe that analyzing USVs in the settings described above may help to study mechanisms underlying ongoing pain, which is one of the major burdens in humans with chronic pain. This is particularly relevant because mechanisms underlying ongoing pain may not overlap with those which mediate evoked hypersensitivity. Furthermore, employing USVs as a parameter in analgesic drug screening may improve the bench-to-bedside translation of novel therapeutics.

## Methods and materials

### Nociceptive tests and mouse models of cancer pain and neuropathic pain

All animal use procedures were in accordance with ethical guidelines imposed by the local governing body (Regierungspräsidium Karlsruhe, Germany). All behavioural measurements were done in awake, unrestrained, age-matched adult (more than 3 months-old) mice of both sexes. Mice were acclimatised to the experimental set-ups several times before the analysis.

Mechanical sensitivity to graded pressure was tested via manual application of von Frey hairs to the plantar surface as described previously [25]. To induce bone metastases, National Collection of Type Cultures (NCTC) clone 2472 fibrosarcoma cells (ATCC) were cultured and injected unilaterally into and around the calcaneus bone in male C3H/HeNCrl mice as described previously [18,19]. The resulting vocalization responses were measured over 12 days in total. Evoked mechanical hypersensitivity cause by the injection of cancer cells was tested 7 and 12 days after the injection using von Frey filaments as described above. In some experiments, mice were tested prior to and 15 minutes after sub-cutaneus injection of fentanyl (0.1 mg/kg) or vehicle.

The Spared nerve injury model was used to study chronic neuropathic pain as described by Decostered and Woolf [20]. Briefly, two of the three terminal branches of the sciatic nerve (tibial and common peroneal nerves) were ligated and cut leaving the remaining third branch (sural nerve) intact. Mechanical hyperalgesia cause by the operation was tested over 8 weeks using von Frey filaments as described previously [25]. Vocalization responses were tested up to 6 weeks after the operation. In some experiments, gabapentin (30 mg/kg) or vehicle was injected intraperitoneally 1 h prior measurement of behavioural responses.

### Data analysis and statistics

All data are presented as mean ± standard error of the mean (S.E.M.). Analysis of Variance (ANOVA) for random measures followed by post-hoc Fisher's test were utilized to determine statistically significant differences (p = 0.05). For data on the number of USVs, statistical comparisons were done on raw data, not on normalized values.

## Competing interests

The authors declare that they have no competing interests.

## Authors' contributions

M.K., U.N., U.H. and S.S. performed research, analyzed data and made the figures. R.K. designed the study, supervised research and wrote the manuscript. All authors read and approved the final manuscript.

## References

[B1] Portenoy RK Lesage P Management of cancer pain Lancet 1999 353 1695 1700 10.1016/S0140-6736(99)01310-0 10335806

[B2] Honore P Mantyh PW Bone cancer pain: from mechanism to model to therapy Pain Med 2000 1 303 9 10.1046/j.1526-4637.2000.00047.x 15101876

[B3] Vickers ER Cousins MJ Neuropathic orofacial pain part 1--prevalence and pathophysiology Aust Endod J 2000 26 19 26 10.1111/j.1747-4477.2000.tb00146.x 11359293

[B4] Jensen TS Gottrup H Sindrup SH Bach FW The clinical picture of neuropathic pain Eur J Pharmacol 2001 429 1 11 10.1016/S0014-2999(01)01302-4 11698022

[B5] King T Vera-Portocarrero L Gutierrez T Vanderah TW Dussor G Lai J Fields HL Porreca F Unmasking the tonic-aversive state in neuropathic pain Nat Neurosci 2009 12 1364 6 10.1038/nn.2407 19783992 PMC3427725

[B6] Ko SW Chatila T Zhuo M Contribution of CaMKIV to injury and fear-induced ultrasonic vocalizations in adult mice Mol Pain 2005 22 1 10 10.1186/1744-8069-1-10 PMC1079936 15813959

[B7] Oliveira AR Barros HM Ultrasonic rat vocalizations during the formalin test: a measure of the affective dimension of pain? Anesth Analg 2006 102 832 9 10.1213/01.ane.0000196530.72813.d9 16492837

[B8] Han JS Bird GC Li W Jones J Neugebauer V Computerized analysis of audible and ultrasonic vocalizations of rats as a standardized measure of pain-related behavior J Neurosci Methods 2005 141 261 9 10.1016/j.jneumeth.2004.07.005 15661308

[B9] Jourdan D Ardid D Chapuy E Le Bars D Eschalier A Effect of analgesics on audible and ultrasonic pain-induced vocalization in the rat Life Sc 1998 63 1761 8 10.1016/S0024-3205(98)00450-0 9820120

[B10] Martino G Perkins MN Tactile-induced ultrasonic vocalization in the rat: a novel assay to assess anti-migraine therapies in vivo Cephalalgia 2008 28 723 33 10.1111/j.1468-2982.2008.01582.x 18498397

[B11] Wallace VC Norbury TA Rice AS Ultrasound vocalisation by rodents does not correlate with behavioural measures of persistent pain Eur J Pain 2005 9 445 52 10.1016/j.ejpain.2004.10.006 15979025

[B12] Jourdan D Ardid D Eschalier A Analysis of ultrasonic vocalisation does not allow chronic pain to be evaluated in rats Pain 2002 95 165 73 10.1016/S0304-3959(01)00394-3 11790479

[B13] Williams WO Riskin DK Mott AK Ultrasonic sound as an indicator of acute pain in laboratory mice J Am Assoc Lab Anim Sci 2008 47 8 10 18210991 PMC2652617

[B14] Wöhr M Borta A Schwarting RK Overt behavior and ultrasonic vocalization in a fear conditioning paradigm: a dose-response study in the rat Neurobiol Learn Mem 2005 84 228 40 10.1016/j.nlm.2005.07.004 16115784

[B15] Litvin Y Blanchard DC Blanchard RJ Rat 22 kHz ultrasonic vocalizations as alarm cries Behav Brain Res 2007 182 166 72 10.1016/j.bbr.2006.11.038 17173984

[B16] Jelen P Soltysik S Zagrodzka J 22-kHz ultrasonic vocalization in rats as an index of anxiety but not fear: behavioral and pharmacological modulation of affective state Behav Brain Res 2003 141 63 72 10.1016/S0166-4328(02)00321-2 12672560

[B17] Blanchard RJ Blanchard DC Agullana R Weiss SM Twenty-two kHz alarm cries to presentation of a predator, by laboratory rats living in visible burrow systems Physiol Behav 1991 50 967 72 10.1016/0031-9384(91)90423-L 1805287

[B18] Cain DM Wacnik PW Turner M Wendelschafer-Crabb G Kennedy WR Wilcox GL Simone DA Functional interactions between tumor and peripheral nerve: changes in excitability and morphology of primary afferent fibers in a murine model of cancer pain J Neurosci 2001 21 9367 76 11717370 10.1523/JNEUROSCI.21-23-09367.2001 PMC6763931

[B19] Schweizerhof M Stösser S Kurejova M Njoo C Gangadharan V Agarwal N Schmelz M Bali KK Michalski CW Brugger S Dickenson A Simone DA Kuner R Hematopoietic colony-stimulating factors mediate tumor-nerve interactions and bone cancer pain Nat Med 2009 15 802 7 10.1038/nm.1976 19525966

[B20] Decosterd I Woolf CJ Spared nerve injury: an animal model of persistent peripheral neuropathic pain Pain 2000 87 149 58 10.1016/S0304-3959(00)00276-1 10924808

[B21] Paix A Coleman A Lees J Grigson J Brooksbank M Thorne D Ashby M Subcutaneous fentanyl and sufentanil infusion substitution for morphine intolerance in cancer pain management Pain 1995 63 263 9 10.1016/0304-3959(95)00084-6 8628593

[B22] El Mouedden M Meert TF The impact of the opioids fentanyl and morphine on nociception and bone destruction in a murine model of bone cancer pain Pharmacol Biochem Behav 2007 87 30 40 10.1016/j.pbb.2007.03.017 17521715

[B23] Jensen TS Madsen CS Finnerup NB Pharmacology and treatment of neuropathic pains Curr Opin Neurol 2009 22 467 74 10.1097/WCO.0b013e3283311e13 19741531

[B24] Omori Y Kagaya K Enomoto R Sasaki A Andoh T Nojima H Takahata H Kuraishi Y A mouse model of sural nerve injury-induced neuropathy: gabapentin inhibits pain-related behaviors and the hyperactivity of wide-dynamic range neurons in the dorsal horn J Pharmacol Sci 2009 109 532 9 10.1254/jphs.08319FP 19346671

[B25] Agarwal N Pacher P Tegeder I Amaya F Constantin C Brenner GJ Rubino T Michalski CW Marsicano G Monory K Mackie K Marian C Batkai S Parolaro D Fischer MJ Reeh P Kunos G Kress M Lutz B Woolf CJ Kuner R Nociceptor-specific conditional gene deletion reveals that cannabinoids mediate analgesia largely via peripheral type 1 cannabinoid receptors Nat Neurosci 2007 10 870 9 10.1038/nn1916 17558404 PMC2234438

